# Crystal structure of CotA laccase complexed with 2,2-azinobis-(3-ethylbenzothiazoline-6-sulfonate) at a novel binding site

**DOI:** 10.1107/S2053230X1600426X

**Published:** 2016-03-24

**Authors:** Zhongchuan Liu, Tian Xie, Qiuping Zhong, Ganggang Wang

**Affiliations:** a Key Laboratory of Environmental and Applied Microbiology, Chengdu Institute of Biology, Chinese Academy of Sciences, Chengdu 610041, People’s Republic of China; b Key Laboratory of Environmental Microbiology of Sichuan Province, Chengdu 610041, People’s Republic of China; c University of Chinese Academy of Sciences, Beijing 100049, People’s Republic of China

**Keywords:** 2,2-azinobis-(3-ethylbenzo­thiazoline-6-sulfonate), laccase, mutagenesis, substrate binding, protein engineering

## Abstract

The CotA laccase from *Bacillus subtilis* is an abundant component of the spore outer coat and has been characterized as a typical laccase. The crystal structure of CotA complexed with 2,2-azinobis-(3-ethylbenzothiazoline-6-sulfonate) (ABTS) in a hole motif has been solved. The novel binding site was about 26 Å away from the T1 binding pocket. Comparison with known structures of other laccases revealed that the hole is a specific feature of CotA. The key residues Arg476 and Ser360 were directly bound to ABTS. Site-directed mutagenesis studies revealed that the residues Arg146, Arg429 and Arg476, which are located at the bottom of the novel binding site, are essential for the oxidation of ABTS and syringaldazine. Specially, a Thr480Phe variant was identified to be almost 3.5 times more specific for ABTS than for syringaldazine compared with the wild type. These results suggest this novel binding site for ABTS could be a potential target for protein engineering of CotA laccases.

## Abstract 2

The crystal structure of CotA complexed with 2,2-azinobis-(3-ethylbenzothiazoline-6-sulfonate) in a hole motif has been solved; this novel binding site could be a potential structure-based target for protein engineering of CotA laccase.

## 1. Introduction   

Spore coat protein A (CotA) is a protein isolated from the bacterium *Bacillus subtilis*. It is able to oxidize specific laccase substrates and is characterized as a typical laccase with one mononuclear copper centre (T1 copper) and one trinuclear centre (TNC) comprised of one T2 copper and two T3 coppers (Bertrand *et al.*, 2002 ▸; Martins *et al.*, 2002 ▸). This enzyme can catalyze the oxidation of a wide variety of substrates by using dioxygen as an electron acceptor. The substrate-binding pocket of CotA is near the T1 copper. Substrate oxidation occurs at the T1 binding pocket and the electrons are then shuttled to the TNC, where the reduction of dioxygen occurs. The water channel is near the T2 copper and points to the protein surface; the water molecules produced by the reduction of dioxygen are released through this channel (Enguita *et al.*, 2003 ▸). As a component of the spore coat, CotA laccase has the dual advantages of both thermostability and thermoactivity. It is believed that a higher proline content and compact packing may be the factors the promote the increased thermostability of CotA (Enguita *et al.*, 2003 ▸). To date, CotA has been used as a model protein in studying the relationship between protein structure and function and in the protein engineering of laccases (Gupta & Farinas, 2010 ▸; Koschorreck *et al.*, 2009 ▸; Mollania *et al.*, 2011 ▸; Jia *et al.*, 2014 ▸; Enguita *et al.*, 2003 ▸, 2004 ▸; Bento *et al.*, 2005 ▸, 2010 ▸; Chen *et al.*, 2010 ▸; Fernandes *et al.*, 2011 ▸; Silva, Damas *et al.*, 2012 ▸; Durão *et al.*, 2008 ▸).

The overall structure of CotA comprises three cupredoxin-type domains, characterized by a Greek-key β-barrel topology, which is similar to other multi-copper oxidases (Messer­schmidt & Huber, 1990 ▸; Messerschmidt *et al.*, 1992 ▸; Zaitseva *et al.*, 1996 ▸). However, structural comparisons reveal that the CotA protein contains some unique structural features. One unique feature is that CotA has a region of positive charge on its surface at the interface between domain 1 and domains 2 and 3 (Enguita *et al.*, 2003 ▸). More than a dozen positively charged residues (ten lysines and five arginines) are located in this area, which leads to a highly positive patch on the surface of CotA. No similar positively charged area has been found in fungal and other bacterial laccases with known structures (Roberts *et al.*, 2003 ▸; Silva, Durão *et al.*, 2012 ▸; Piontek *et al.*, 2002 ▸; Garavaglia *et al.*, 2004 ▸). The biological significance of this positively charged region is not known, but it has been speculated that it may be involved in the assembly of CotA into the spore outer coat layer (Enguita *et al.*, 2003 ▸; McKenney *et al.*, 2013 ▸; Bauer *et al.*, 1999 ▸).

Another interesting feature of CotA is located close to the positively charged region of the protein. An extruding loop comprising residues 359–365 and adjacent residues form a hole near to the water channel. The positive residues Arg146, Arg429 and Arg476 are located on one side of the hole. The significance of this hole is currently unknown, but it has been reported that a mutation of Thr480 to alanine at the entrance to the hole results in increased activity of CotA on the spore surface (Jia *et al.*, 2014 ▸). Therefore, it is of interest to investigate the properties of this hole in CotA.

Here, we report the crystal structure of CotA complexed with a nonphenolic substrate, 2,2-azinobis-(3-ethylbenzothiazoline-6-sulfonate) (ABTS). The ABTS molecule unexpectedly binds to the positively charged hole in the protein. The novel substrate-binding site is described and the biological significance of this motif is discussed.

## 2. Materials and methods   

### 2.1. Protein crystallization and data collection   

Purification of recombinant CotA and the mutant proteins was performed as described previously (Xie *et al.*, 2015 ▸). Crystals of the CotA proteins were obtained at 18°C by the vapour-diffusion method from a reservoir solution consisting of 30–42%(*v*/*v*) ethylene glycol, 100 m*M* sodium citrate pH 5.6 (Table 1 ▸). Crystals were grown over a period of 5–7 d and were then soaked in a solution consisting of 5 m*M* ABTS for approximately 20 s. It was observed that the colour of the crystal changed from light blue to dark green. The crystals were then transferred into a cryoprotectant solution consisting of 25%(*v*/*v*) glycerol in the crystal mother liquor before being flash-cooled in liquid nitrogen. Diffraction data were collected on the 3W1A beamline at the Beijing Synchrotron Radiation Facility (BSRF). Data sets were processed and scaled using *HKL*-2000 (Otwinowski & Minor, 1997 ▸) (Table 2 ▸). All crystals were flash-cooled and maintained at 100 K in a flow of cold nitrogen gas during data collection.

### 2.2. Structure determination and refinement   

The structures of native CotA and of the CotA–ABTS complex were elucidated by molecular replacement using *Phaser* (McCoy *et al.*, 2007 ▸) from the *CCP*4 program suite (Winn *et al.*, 2011 ▸). The starting model was the native structure of CotA (PDB entry 1gsk; Enguita *et al.*, 2003 ▸), from which the solvent molecules, as well as the copper ions, had been removed. Only one solution was evident. Refinement of the structural model was completed using the maximum-likelihood functions implemented in *REFMAC*5 (Murshudov *et al.*, 2011 ▸) from the *CCP*4 program suite. Model building and improvement was performed with *Coot* (Emsley *et al.*, 2010 ▸) in combination with σ_A_-weighted 2*F*_o_ − *F*_c_ and *F*_o_ − *F*_c_ maps. Subsequent electron-density calculation enabled the location of three of the four copper ions in the native and complex structures. No obvious positive peaks for the T2 copper were observed in the *F*_o_ − *F*_c_ maps at a σ level of 1.0 for the native protein or its ABTS complex; therefore, the T2 copper was not included in the final models. The ABTS molecule was included in the complex after a few refinement cycles because of clear density in both 2*F*_o_ − *F*_c_ and *F*_o_ − *F*_c_ maps. After these rounds of refinement, solvent molecules were added to the models based on standard geometrical and chemical restraints. The occupancies of the copper ions were refined by *REFMAC*5 individually. Residues 91–95 in the native structure and residues 91–94 in the complex structure were not built in the final models because of the poor quality of the electron density. The initial methionine and the final two residues in both structures were disordered and were not included. *PROCHECK* (Laskowski *et al.*, 1993 ▸) was used for validation. Details of the overall refinement and final quality of the models are shown in Table 3 ▸. *PyMOL* (http://www.pymol.org) was used to prepare structural figures.

### 2.3. Enzyme assay   

The laccase activity towards ABTS and syringaldazine (SGZ) was measured at 40°C. The ABTS assay mixture contained 1 m*M* ABTS and 100 m*M* citrate–phosphate buffer pH 4.0. Oxidation of ABTS was followed by the increase in absorbance at 420 nm (∊_420_ = 36 000 *M* ^−1^ cm^−1^). The SGZ assay mixture contained 0.1 m*M* SGZ (dissolved in ethanol) and 100 m*M* citrate–phosphate buffer pH 7.0. Oxidation of SGZ was followed by the increase in absorbance at 525 nm (∊_525_ = 65 000 *M* ^−1^ cm^−1^). The protein concentration was determined by the Bradford method (Bradford, 1976 ▸) using bovine serum albumin as the standard. All assays were performed in triplicate to calculate the standard error.

### 2.4. Solvent-accessible surface calculation   

Calculation of the solvent-accessible surface was performed using the *CASTp* server (Dundas *et al.*, 2006 ▸).

### 2.5. Copper-content determination   

To determine the copper content of CotA proteins, 500 µl of a solution containing 5 mg ml^−1^ protein was mixed with 500 µl nitric acid and hydrolyzed at 100°C for 2 h. The sample was diluted to a suitable volume and measured using an atomic absorption spectrometer (PinAAcle 900T, PerkinElmer).

## 3. Results and discussion   

### 3.1. The hole structure in CotA laccase   

**Figure 1:**
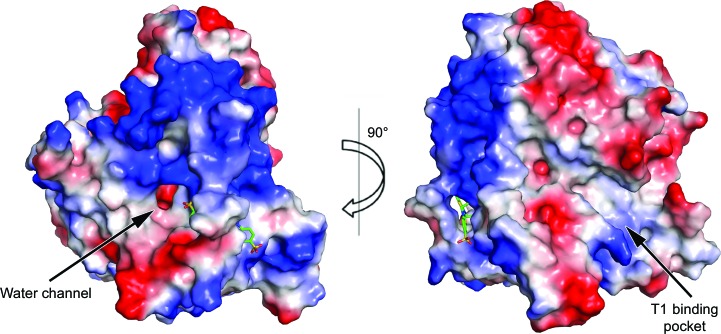
Surface electrostatic potential of CotA. A region with a large positive potential was found in the connection area between domains. The water channel and the T1 binding pocket are labelled. The ABTS molecule is represented as a green stick model.

Crystal structures of native CotA and the CotA–ABTS complex have been determined at 2.3 Å resolution. The asymmetric units of the crystals contained one molecule in space group *P*3_1_21. At the interface between domain 1 and domains 2 and 3 there was a positively charged surface region. Moreover, the linking loop (residues 359–365) between domains 2 and 3 and four β-strands of domain 3 formed a hole with dimensions of 8 × 5 × 4 Å and a calculated surface area of 159 Å^2^. In the structure of the CotA–ABTS complex, ABTS was observed sitting within the hole. The novel binding site was approximately 26 Å away from the T1 binding pocket and was near the exit of the water channel (Fig. 1 ▸).

**Figure 2:**
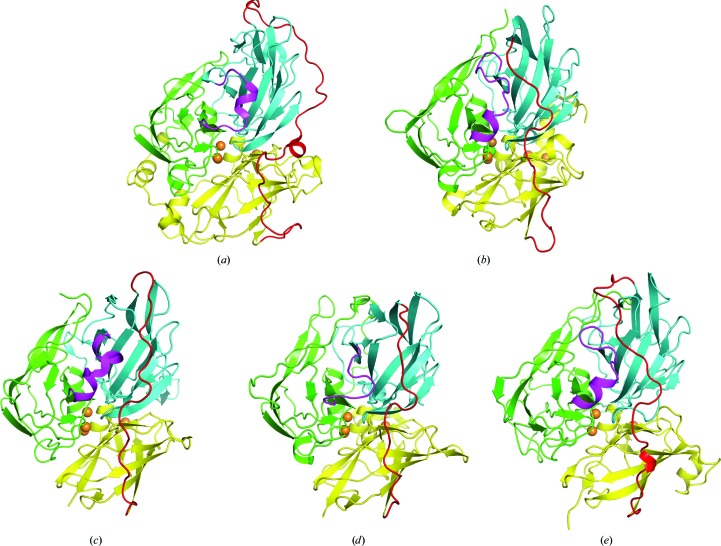
Crystal structures of laccases. (*a*) Tvl from *T. versicolor* (PDB entry 1gyc; Piontek *et al.*, 2002 ▸), (*b*) CueO from *E. coli* (PDB entry 1n68; Roberts *et al.*, 2003 ▸), (*c*) Thhb27 from *Thermus thermophilus* (PDB entry 2xu9; Serrano-Posada *et al.*, 2015 ▸), (*d*) McoC from *Campylobacter jejuni* CGUG11284 (PDB entry 3zx1; Silva, Durão *et al.*, 2012 ▸), (*e*) CotA from *B. subtilis*. Domains 1, 2 and 3 are coloured green, blue and yellow, respectively. Cu atoms are represented as spheres and are coloured orange. The linking loop between domains 2 and 3 is coloured red and the peptide connection between domains 1 and 2 is coloured magenta.

Structural comparisons were performed to determine whether fungal and bacterial laccases contained a similar hole. In the fungal laccase Tvl from *Trametes versicolor* (Piontek *et al.*, 2002 ▸) there was a 40-residue extended loop region between domains 2 and 3, which is approximately ten residues longer than that in the bacterial laccase. While the 40-residue loop in Tvl laccase wound from the side of the β-barrel domains *via* an internal connection, this connection did not result in the formation of a hole in the fungal laccase. This loop was clearly distant from the 3_10_-helical fragment between domains 1 and 2 (Fig. 2 ▸ *a*). However, the equivalent loop in the bacterial laccase spanned over the β-barrel by an external connection (Figs. 2 ▸ *b*–2 ▸ *e*), which may be a characteristic of prokaryotic enzymes.

**Figure 3:**
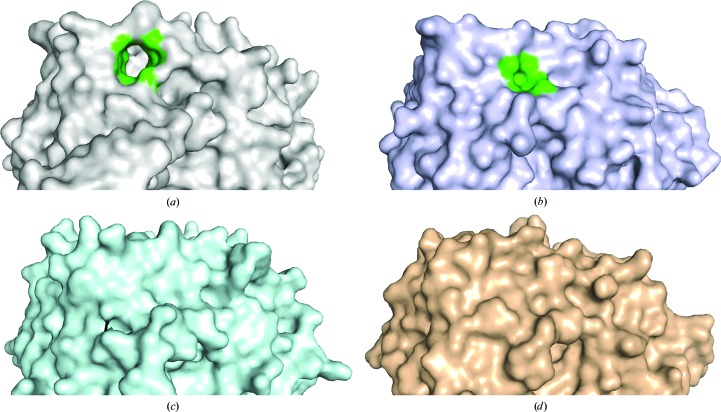
Molecular-surface representation and the putative cavity adjacent to the linking loop between domains 2 and 3. (*a*) CotA, (*b*) CueO, (*c*) McoC, (*d*) Thhb27. The surface is coloured grey for CotA, blue-white for CueO, cyan for McoC and orange for Thhb27. All molecular-surface representations are in the same orientation. The cavity is coloured green; the other cavity of CueO is on the opposite side and is not visible in this orientation.

Moreover, cavity analysis was performed on CotA and another three bacterial laccases (Fig. 3 ▸). In CotA, the predicted hole was exactly the same as that in the crystal structure. In CueO (Roberts *et al.*, 2003 ▸) two cavities were found adjacent to the linking loop; however, the cavities were only open on one side and the surface areas of the two cavities were 11 and 4 Å^2^, respectively. In both McoC and Thhb27 (Silva, Durão *et al.*, 2012 ▸; Serrano-Posada *et al.*, 2011 ▸) no obvious cavity was formed by the corresponding loop. The loop linking domains 2 and 3 in CotA was 27 residues in length, which is longer than those in CueO (20 amino acids), McoC (19 amino acids) and Thhb27 (22 amino acids). This may lead to the loose connection between domains 2 and 3 in CotA and favour formation of the hole.

**Figure 4:**
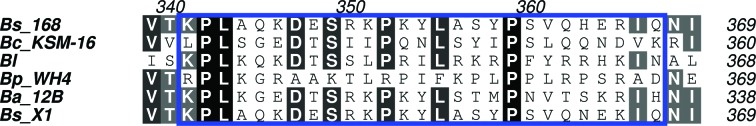
Multiple sequence alignment of the loop region linking domains 2 and 3 of CotA laccase. The linking loop is boxed in blue. *Bs_168*, *B. subtilis* 168; *Bc_KSM-K16*, *B. clausii* KSM-K16; *Bl*, *B. licheniformis*; *Bp-WH4*, *B. pumilus* WH-4; *Ba_12B*, *B. amyloliquefaciens* 12B; *Bs_X1*, *B. subtilis* X1.

Laccases are multi-copper oxidases that are widely distributed in nature. Structural studies revealed that laccases possess conserved copper-ion centres and overall topology. In contrast, it was reported that the laccases from various species contained specific features. A conserved C-terminal end (DSGL) was observed in the laccase from *Melanocarpus albomyces*, which penetrated the tunnel leading from the protein surface to the TNC (Andberg *et al.*, 2009 ▸). A laccase from silkworm (*Bombyx mori*) was identified as a dimer, containing an extra domain of approximately 200 amino acids that was not present in laccases from plants and fungi (Dittmer *et al.*, 2004 ▸). CueO from *Escherichia coli* contained a fifth labile copper bound to a methionine-rich motif. This labile copper could mediate electron transfer from the substrate to the T1 copper (Roberts *et al.*, 2002 ▸). As a component of the spore-coat outer layer from *B. subtilis*, CotA may also exhibit specific features (McKenney *et al.*, 2013 ▸). The sequences of CotA from strains of *Bacillus* are highly conserved (Xie *et al.*, 2015 ▸). Therefore, all of the CotA enzymes could have a similar structure. Multiple sequence alignment shows that the lengths of the loops linking domains 2 and 3 in the CotA proteins are similar to each other (Fig. 4 ▸) and, moreover, the hole structure of CotA was not found in the fungal laccase or other bacterial laccases; therefore, this hole structure could be a specific feature of the CotA protein from strains of *Bacillus*.

### 3.2. Novel binding site and conformational changes induced by the binding of ABTS   

**Figure 5:**
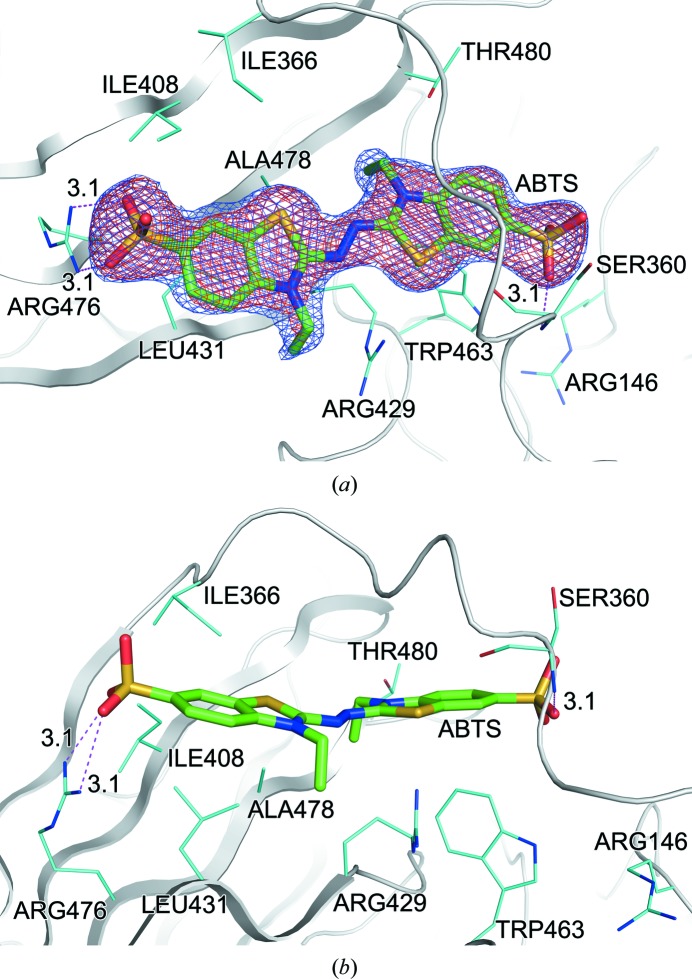
CotA–ABTS interactions. Residues in close proximity to ABTS are labelled and represented as cyan stick models showing (*a*) the top view and (*b*) the side view. The 2*F*_o_ − *F*_c_ electron-density map (1σ level) around ABTS is represented in blue and the OMIT *F*_o_ − *F*_c_ map (4σ level) around ABTS is represented in red. The ABTS molecule is shown as a green stick model.

**Figure 6:**
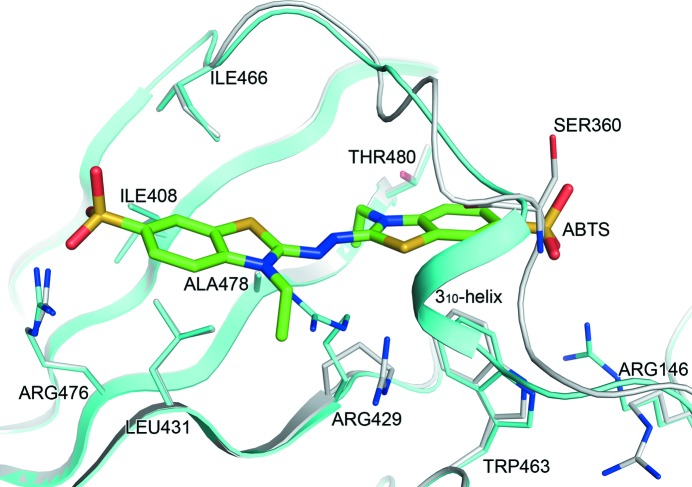
Superposition of the CotA–ABTS complex (grey) onto native CotA (cyan). Residues in close proximity to ABTS are labelled and represented as stick models. The ABTS molecule is represented as a green stick model.

The ABTS molecule was identified from an interpretable electron density wrapped by the loop region of residues 359–365 (Fig. 5 ▸). This novel binding site was mainly formed by the extruding loop segment and four β-stands of domain 3. The bottom of the hole was positively charged owing to residues Arg146, Arg429 and Arg476. The ABTS molecule passed through the hole in a linear shape and interacted with adjacent residues by hydrophobic contacts: Ile408, Leu431, Ile366, Ala478 and Trp463. Moreover, the ABTS was directly bound to Arg476 and Ser360. The sulfonate moiety at one end of ABTS formed two hydrogen bonds to the guanidyl group of Arg476 (Arg476 NH1–O48 = 3.1 Å, Arg476 NH2–O49 = 3.1 Å), and the sulfonate moiety at the other end of ABTS formed a hydrogen bond to Ser360 (Ser360 N–O45 = 3.1 Å). The thiazoline ring of ABTS was almost buried in the hole, and the two sulfonate moieties of ABTS were exposed to the external environment. In the CotA–ABTS complex, conformational changes were observed upon substrate binding. A 3_10_-helical fragment composed of residues 359–362 was dissolved and the side chains of Arg429 and Arg146 were rotated by about 40 and 120°, respectively, providing more space for ABTS binding (Fig. 6 ▸).

In a previous study, ABTS bound in the T1 binding pocket as a shallow U-shaped molecule with an overall *B* factor of 55.1 Å^2^ and 40% occupancy. Only one half of the ABTS molecule was partially buried in the CotA structure (Enguita *et al.*, 2003 ▸, 2004 ▸). In our study, the ABTS passed through the hole in a linear shape; the *B* factor of ABTS was 35.6 Å^2^ and it had full occupancy. Compared with the U-shaped binding state, the binding of ABTS to CotA described here was more stable.

In this work, the CotA crystal was soaked in 5 m*M* ABTS for 20 s. The use of a high concentration of ABTS meant that the molecule was more easily able to access the hole motif. In addition, the interactions between ABTS and the adjacent residues in the hole stabilized the binding of ABTS without any oxidation occurring. However, in the T1 binding pocket oxidation proceeded rapidly, which resulted in the product being released. It is noted that the shape of the T1 binding pocket continuously changed, making it hard to form a stable complex. In the previous report, the CotA crystal was soaked in 0.5 m*M* ABTS for 1 h. The lower substrate concentration and longer soaking time used in that research may account for the difference, as after 1 h of soaking the ABTS was already oxidized, meaning that the ABTS was bound in its cationic state (Enguita *et al.*, 2004 ▸).

### 3.3. Kinetic characterization of CotA and mutants   

A group of residues (Arg146, Arg429, Arg476, Leu431, Ala478 and Thr480) in the novel binding site have been mutated. The substrates ABTS and SGZ were used to study the catalytic properties of the mutants. The data for the kinetic parameters are shown in Table 4 ▸. The copper contents of wild-type CotA and five mutants, R146K, R429K, A478F, T480A and T480F, were also measured, as shown in Table 5 ▸. A stoichiometry of close to four coppers per protein was calculated for both mutant and wild-type CotA, showing that all four copper ions were incorporated into the active site. Since the mutation sites were distant from the copper-coordinating sites, the mutation may not affect the stability of the Cu atoms; this may also be the case for the L431F and R476K mutants. 

In our work, substitution of arginine by lysine in the novel binding cavity dramatically affected the activity of the CotA enzyme, and the decrease in activity was not substrate-dependent. The activity of the R146K and R429K mutants decreased 30–100-fold in efficiency (*k*_cat_/*K*_m_) compared with the wild type (WT). The R476K mutant retained approximately 20% of the WT activity with both ABTS and SGZ. The L431F, T480A and A478F mutants displayed approximately 50, 60 and 70% of the activity of the WT, respectively. Moreover, the T480F mutant exhibited different effects in the oxidation of ABTS and SGZ. The T480F mutant showed equivalent activity compared with the WT in the oxidation of ABTS, whereas only about 30% of the activity of the WT was retained in the oxidation of SGZ. To assess substrate specificity, (*k*_cat_/*K*_m_)_ABTS_/(*k*_cat_/*K*_m_)_SGZ_ was calculated. The ratios for wild-type CotA and the T480F mutant were approximately 0.82 ± 0.03 and 2.90 ± 0.09, respectively. Therefore, the T480F mutant was almost 3.5 times more specific for ABTS than for SGZ compared with the WT. It was reported that a T480A mutant of CotA displayed on the surface of the bacterium spore was 2.38-fold more active compared with WT CotA in organic solvent (Jia *et al.*, 2014 ▸). In our work, the T480A mutant showed 60% of the activity compared with the WT in a buffer solution. This may be because of structural differences between the enzyme in solution and the enzyme displayed on the spore surface.

All of these residues except Arg146 are located in domain 3 of CotA. This domain not only contains the mononuclear copper centre but also contributes to the formation of the binding site of the TNC. Mutations in the specific residues that compose the novel binding cavity may contribute to changes at the catalytic centre of CotA; this was also reported for various mutant sites located in domain 3, although these sites were distant from the copper centres (Gupta & Farinas, 2010 ▸; Bao *et al.*, 2013 ▸; Kenzom *et al.*, 2014 ▸; Jia *et al.*, 2014 ▸).

Over the years, laccases have become attractive candidates for protein engineering. Various CotA mutants have been prepared by rational, semi-rational or directed evolution approaches, and biocatalysts with improved features or new functions were obtained. A mutant of CotA from *Bacillus* sp. HR03 exhibited higher thermal stability (Mollania *et al.*, 2011 ▸), and the substrate specificity of CotA was narrowed by saturation mutagenesis (Gupta & Farinas, 2009 ▸, 2010 ▸). In the *B. subtilis* spore, the CotA-ABTS-SD1 mutant (T260L) exhibited 120-fold higher specificity for ABTS compared with the WT (Gupta & Farinas, 2010 ▸). In addition, the double mutant K316N/D500G of CotA from *B. licheniformis* displayed a 11.4-fold higher expression level in *E. coli* compared with the WT (Koschorreck *et al.*, 2009 ▸). Here, a novel binding site in CotA was revealed, with the CotA mutants described above showing different effects on the oxidation of ABTS and SGZ. Specially, the T480F mutant was found to be almost 3.5 times more specific for ABTS than for SGZ compared with the WT. This implies that rational design could be performed on the novel binding site to improve some of the properties of CotA proteins.

## 4. Conclusions   

In summary, the hole motif described in this paper is a specific feature of CotA proteins into which the substrate ABTS can bind. This novel binding site could be a potential structural target for protein engineering of CotA laccases.

## Supplementary Material

PDB reference: CotA, wild type, 4yvu

PDB reference: complex with ABTS, 4yvn

## Figures and Tables

**Table 1:** Crystallization

Method	Vapour diffusion
Plate type	Hanging drop, 24-well
Temperature (K)	291
Protein concentration (mg ml^−1^)	6
Buffer composition of protein solution	100 m*M* sodium citrate pH 5.6
Composition of reservoir solution	30–42%(*v*/*v*) ethylene glycol
Volume and ratio of drop	2 µl, 1:1
Volume of reservoir (µl)	200

**Table 2:** Data collection and processing Values in parentheses are for the outer shell.

	ABTS complex	Native
Diffraction source	3W1A, BSRF	3W1A, BSRF
Wavelength (Å)	1.0	1.0
Temperature (K)	100	100
Detector	MAR CCD 165 mm	MAR CCD 165 mm
Crystal-to-detector distance (mm)	150	150
Rotation range per image (°)	1	1
Total rotation range (°)	140	120
Exposure time per image (s)	15	25
Space group	*P*3_1_21	*P*3_1_21
*a*, *b*, *c* (Å)	101.99, 101.99, 135.79	101.73, 101.73, 135.57
α, β, γ (°)	90, 90, 120	90, 90, 120
Mosaicity (°)	0.296	0.375
Resolution range (Å)	29.4–2.30 (2.34–2.30)	29.4–2.30 (2.34–2.30)
Total No. of reflections	160013 (7883)	135883 (6623)
No. of unique reflections	36864 (1811)	36605 (1784)
Completeness (%)	99.9 (100)	100 (100)
Multiplicity	4.3 (4.4)	3.7 (3.7)
〈*I*/σ(*I*)〉	9.6 (6.5)	12.2 (4.6)
*R*_merge_ (%)	14.7 (25.5)	9.7 (28.1)
Overall *B* factor from Wilson plot (Å^2^)	34.9	35.0

**Table 3:** Structure solution and refinement Values in parentheses are for the outer shell.

	ABTS complex	Native
Resolution range (Å)	29.46–2.30 (2.360–2.300)	29.38–2.30 (2.360–2.300)
No. of reflections, working set	34956 (2584)	34656 (2473)
No. of reflections, test set	1837 (121)	1824 (108)
Final *R*_cryst_	0.158 (0.186)	0.159 (0.185)
Final *R*_free_	0.194 (0.217)	0.187 (0.205)
No. of non-H atoms		
Protein	4070	4080
Ion	3	3
Ligand	58	22
Solvent	394	405
Total	4525	4517
R.m.s. deviations		
Bonds (Å)	0.015	0.014
Angles (°)	1.647	1.594
Average *B* factors (Å^2^)		
Protein	21.7	21.0
Ion	15.2	14.8
Ligand	33.4	29.7
Water	28.8	30.3
Ramachandran plot		
Most favoured (%)	96.2	96.6
Allowed (%)	3.8	3.4

**Table 4:** Kinetic constants of CotA and its mutants for ABTS and SGZ

	ABTS			SGZ			
Enzyme	*K*_m_ (µ*M*)	*k*_cat_ (s^−1^)	*k*_cat_/*K*_m_ (µ*M* ^−1^ s^−1^)	*K*_m_ (µ*M*)	*k*_cat_ (s^−1^)	*k*_cat_/*K*_m_ (µ*M* ^−1^ s^−1^)	(*k*_cat_/*K*_m_)_ABTS_/(*k*_cat_/*K*_m_)_SGZ_
WT	105.51 ± 19.32	51.82 ± 3.85	0.50 ± 0.05	25.78 ± 4.54	15.48 ± 1.41	0.61 ± 0.05	0.82 ± 0.03
R146K	257.66 ± 3.8	0.36 ± 0.01	0.0014 ± 0.0008	13.45 ± 3.43	0.05 ± 0.005	0.004 ± 0.001	0.35 ± 0.09
R429K	90.61 ± 16.8	1.71 ± 0.23	0.02 ± 0.001	7.44 ± 1.67	0.14 ± 0.01	0.02 ± 0.003	1.00 ± 0.07
R476K	101.03 ± 19.03	11.31 ± 2.49	0.11 ± 0.003	29.32 ± 6.21	4.27 ± 0.68	0.15 ± 0.001	0.73 ± 0.01
L431F	77.68 ± 9.34	20.93 ± 2.91	0.27 ± 0.01	32.93 ± 3.49	8.08 ± 1.87	0.25 ± 0.08	1.08 ± 0.03
A478F	109.88 ± 15.24	40.22 ± 4.67	0.37 ± 0.01	32.71 ± 7.20	12.53 ± 3.07	0.38 ± 0.05	0.97 ± 0.03
T480A	110.09 ± 22.48	35.88 ± 6.18	0.33 ± 0.03	37.40 ± 6.37	12.92 ± 2.96	0.34 ± 0.04	0.97 ± 0.05
T480F	52.95 ± 8.56	29.33 ± 5.89	0.53 ± 0.08	40.03 ± 6.76	7.36 ± 2.00	0.18 ± 0.03	2.90 ± 0.09

**Table 5:** Copper content in wild-type and mutant CotA

Protein	Copper content (moles of Cu/moles of protein)
WT	4.51 ± 0.02
R146K	3.71 ± 0.03
R429K	4.52 ± 0.02
A478F	3.78 ± 0.06
T480A	4.19 ± 0.05
T480F	3.83 ± 0.06
